# Inflammatory biomarkers and delirium: a Mendelian randomization study

**DOI:** 10.3389/fnagi.2023.1221272

**Published:** 2023-08-15

**Authors:** Miao Yu, Yuxuan Li, Baohua Li, Qinggang Ge

**Affiliations:** ^1^Department of Nursing, Peking University Third Hospital, Beijing, China; ^2^Department of Intensive Care Unit, Peking University Third Hospital, Beijing, China

**Keywords:** delirium, inflammatory markers, Mendelian randomization, summary genome-wide association study, IL-6

## Abstract

**Background:**

The association between inflammatory biomarkers and individual delirium symptoms remains controversial in observational studies. We investigated the relationship between inflammatory biomarkers and the risk of developing delirium.

**Methods:**

A bidirectional two-sample Mendelian randomization (MR) was performed. Genetic instruments associated with peripheral tumor necrosis factor-a (TNF-a) C-reactive protein (CRP), interleukin (IL)-1α, IL-1β, IL-2, IL-8, IL-6, soluble IL-6 receptor alpha (sIL-6Rα), and soluble gp130 were identified in three different large summary genome-wide association studies (GWAS) conducted in the European population. Summary-level statistics for delirium not induced by alcohol and other psychoactive substances were obtained from the FinnGen consortium (2,612 cases and 325,306 controls). The estimated causal effects were performed using instruments' variants at the genome-wide significant level (*P* < 5e-8 and *P* < 5e-6), applying a linkage disequilibrium clumping approach with a threshold of *r*^2^ < 0.001 for each of the exposures. Reverse causation was also performed. The inverse-variance weighted method (IVW), MR-Egger method, weighted median method, MR-Egger regression, and MR Pleiotropy RESidual Sum were used for MR analyses.

**Results:**

At the genome-wide significant level (*P* < 5e-8, *r*^2^ < 0.001), genetically predicted sIL-6Rα was significantly associated with a decreased risk of delirium with less than three single-nucleotide polymorphisms (SNPs) in all three GWAS data sources (OR_Waldratio_ = 0.89, 95% CI: 0.79–0.96, *P*_Waldratio_ = 0.0016; OR_IVW_ = 0.88, 95% CI: 0.79–0.97, *P*_IVW_ = 0.008; OR_IVW_ = 0.88, 95% CI: 0.80–0.96, *P*_IVW_ = 0.004). The causal relationship between sIL-6Rα and delirium became non-significant when a more liberal threshold of *P* of < 5e-6 was applied (all *P*_IVW_ > 0.05). At the two genome-wide significance levels (*P* < 5e-8 and *P* < 5e-6), we found no evidence for the causal effects of peripheral TNF-α, CRP, IL-1α, IL-1β, IL-2, IL-6, IL-8, and soluble gp130 on delirium (all *P* > 0.05). The MR-Egger intercept and MR-PRESSO results indicated that no SNP had possible pleiotropy (all *P* > 0.05). Regarding the reverse, no evidence for an effect of delirium on these inflammatory biomarkers could be found (all *P* > 0.05).

**Conclusion:**

The results of this MR analysis did not support that peripheral TNF-α, CRP, IL-1α, IL-1β, IL-2, IL-6, sIL-6Rα, soluble gp130, and IL-8 were causally associated with delirium. More research is needed to explore the role of inflammatory factors in the pathogenesis of delirium.

## 1. Introduction

Delirium is a serious and common neuropsychiatric syndrome in the elderly, characterized by acute and reversible inattention and cognitive dysfunction (Inouye, 2006), resulting in prolonged hospital stays (Zhang et al., 2022), increased hospitalization mortality (Tieges et al., 2021), and long-term cognitive impairment (Goldberg et al., 2020). Despite this, the pathophysiological mechanism of delirium remains unclear, and there is no effective intervention to treat delirium, making its management challenging (Oh et al., 2017). One of the leading pathophysiological hypotheses is that the activation of peripheral pro-inflammatory cytokines causes damage to the blood–brain barrier (Dunne et al., 2021). Identifying associated inflammatory biomarkers may provide potential prevention or treatment strategies for delirium and improve patient outcomes.

Although some inflammatory biomarkers have been associated with delirium in prospective studies and meta-analyses, the results of studies have been controversial. A meta-analysis has shown a modest association between delirium and serum interleukin (IL)-1 receptor antagonist and IL-6 (Wang et al., 2021). High serum IL-6 or C-reactive protein (CRP) levels were reported to increase the risk of postoperative delirium (POD) in three meta-analyses of cohort or case–control studies. The effects of serum IL-8, IL-10, and tumor necrosis factor-α (TNF-α) on the risk of POD were lost in pooled analyses (Liu et al's., 2018; Noah et al., 2021; Huang et al., 2022). Conversely, in a recent prospective cohort study, multivariate logistic regression analysis showed that perioperative increases in plasma IL-10 and neutrophil gelatin enzyme-related lipids were associated with POD, but CRP, IL-1β, and IL-6 were not (Yang et al., 2022). The conflicting results mentioned above may be due to the fact that observational studies are particularly vulnerable to bias caused by unmeasured confounding and reverse causation. Whether inflammatory factors cause delirium remains unclear and needs to be further investigated with an alternative method.

Mendelian randomization (MR) is a useful method for determining the causality of exposures and outcomes because it uses genetic variants as instruments' variables to reveal the effect of exposures on the outcomes of interest (Bowden and Holmes, 2019). Based on the random allocation of alleles, the MR analysis addresses the limitation of conventional studies, such as confounding and reverse causation bias. Few studies have evaluated the causal links between inflammatory factors and delirium using MR analyses. At the same time, considering the high cost of time, labor, and money, randomized controlled trials were scarce. In this study, we performed a bidirectional two-sample MR analysis based on summary-level data of genome-wide association studies (GWAS) to explore the effect of nine commonly mentioned peripheral inflammatory factors [TNF-α, CRP, IL-1α, IL-1β, IL-2, IL-6, soluble IL-6 receptor alpha (sIL-6Rα), soluble gp130, and IL-8] on the risk of delirium.

## 2. Materials and methods

### 2.1. Study design

**Figure 1:**
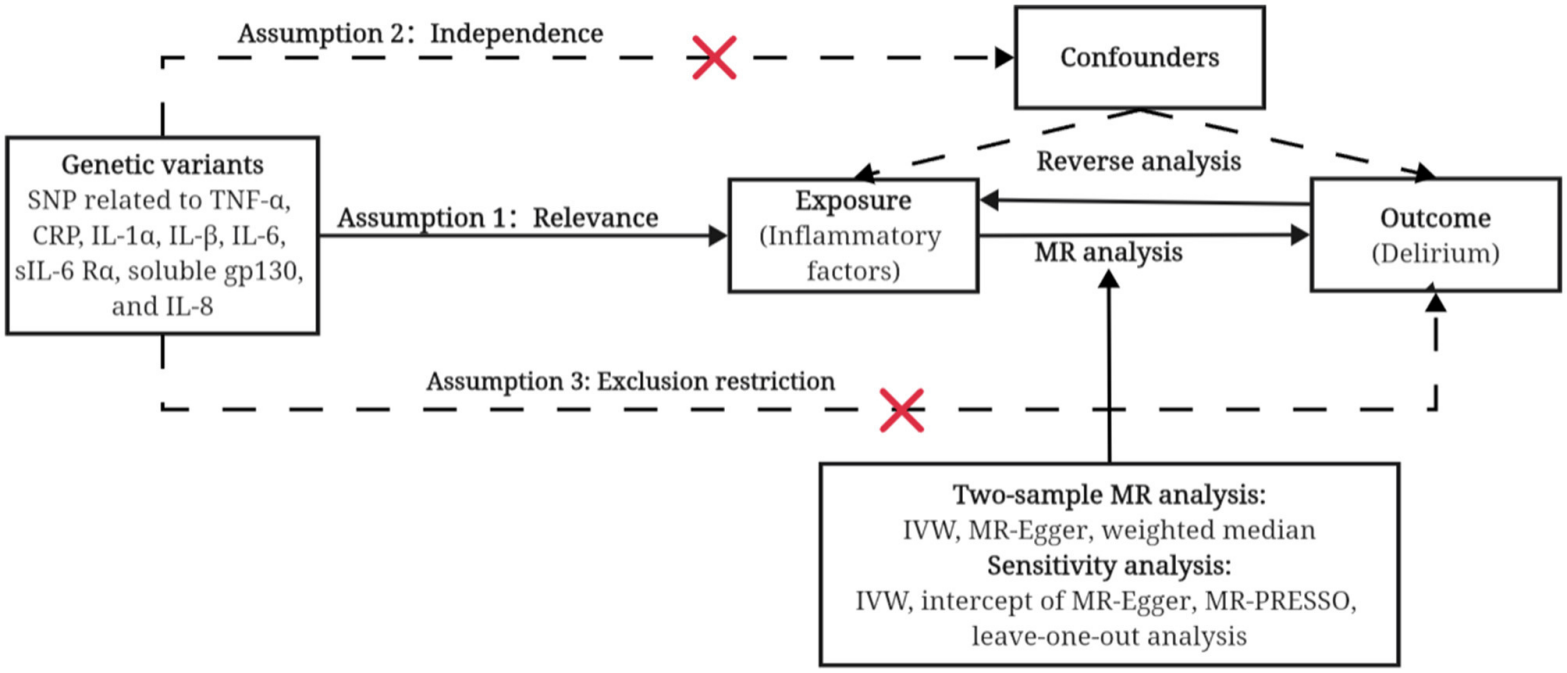
An overview of the study design. Assumption 1: Relevance, instrument variants must be independent of each other and robustly associated with exposure. Assumption 2: Independence, instrument variants must be not associated with confounders between cytokines and delirium. Assumption 3: Exclusion restriction, instrument variants have no direct effects on the outcome, i.e., must go via the exposure. SNP, single-nucleotide polymorphism; MR, Mendelian randomization; IVW, inverse-variance weighted; MR-PRESSO, Mendelian randomization-pleiotropy residual sum and outlier.

The design of our study is shown in Figure 1. Ethical approval was not applicable in this study as data utilized here were freely available for public use. This study was reported in accordance with the Strengthening the Reporting of Observational Studies in Epidemiology Using Mendelian Randomization (STROBE-MR) reporting guideline (Skrivankova et al., 2021).

### 2.2. GWAS data source

Summary-level statistics for delirium that were not induced by alcohol and other psychoactive substances were obtained from the FinnGen consortium (round 8, https://www.finngen.fi/en), comprising 2,612 cases and 325,306 controls (100% of European ancestry). Delirium was diagnosed using the International Statistical Classification of Diseases (ICD) codes (ICD-10: F05 and ICD-9: 2930).

Detailed descriptions for inflammatory biomarkers are shown in Table 1 and Supplementary material. The biomarkers were selected based on a literature review (Dunne et al., 2021) and included TNF-α, CPR, IL-1α, IL-1β, IL-2, IL-6, sIL-6Rα, Soluble gp130, and IL-8. We obtained genetic data from three GWAS studies to support the robustness of MR results. The biomarkers measured in the studies conducted by Suhre et al. and Sun et al. were obtained from blood plasma samples, whereas the biomarkers examined in the study conducted by Gilly et al. were obtained from blood serum samples. These GWAS studies were selected because the samples were restricted to European descent and did not include Finland participants.

**Table 1:** GWAS data sources used in the analysis.

**GWAS data source**	**Measurement or diagnostic criteria**	**Countries sampled (number of participants)**	**Covariates**
Suhre et al. (2017)	SOMAscan assay; relative fluorescent units	Germany (*n* = 997)	Linear regression on inverse-normalization adjusting for age, gender, and body mass index
Sun et al. (2018)	SOMAscan assay; relative fluorescent units	The United Kingdom (*n* = 3,301)	Linear regression on rank-based inverse normal transformation adjusting for age, gender, and body mass index
Gilly et al. (2020)	Olink Proximity Extension Assay CVDII, CVDIII, and metabolism panels; relative quantification units	Greece (*n* = 1,328)	Linear regression on inverse-normal transformation adjusting for age, age squared, gender, plate number, and mean. Normalized protein expression level across all proteins, per sample, and season

### 2.3. Instruments' variant selection

To satisfy the three key assumptions of MR analysis, we selected the single-nucleotide polymorphisms (SNPs) for inflammatory factors if they met the following criteria (Bowden and Holmes, 2019): (1) Instruments' variants must be independent of each other and robustly associated with exposure. Since all inflammatory biomarkers had less than five significant SNPs at the genome-wide significance level (*P* < 5e-8), we also used a liberal significance threshold (*P* < 5e-6). A linkage disequilibrium clumping approach based on *r*^2^ < 0.001 was applied within a 10,000 kb window assessed in the European 1,000 genomes reference panel; (2) instruments' variants must not be associated with confounders between cytokines and delirium. Once potential confounders were detected, the corresponding SNPs were removed; and (3) instruments' variants have no direct effects on the outcome, i.e., they must only be associated with the outcome *via* the exposure. We discarded those absent in the outcome without appropriate proxies SNPs identified by R software. Finally, we used *F*-statistics ($\beta_{e}{}_{\text{xposure}}^{2}$/SE${}_{\text{exposure}}^{2}$) to quantify the strength of the selected SNPs.

### 2.4. Reverse analysis

To examine a possible reverse causal association between delirium and its impact on inflammatory factors, we repeated the steps of the genetic variant selection method to select for delirium instruments' variables. Owing to the limited number of SNPs, we extracted SNPs associated with delirium at the genome-wide significance level (*P* < 5e-6) and LD based on *r*^2^ < 0.001. The SNPs genetically related to delirium (exposure) and inflammatory factors (outcomes) were extracted from the same GWAS mentioned earlier. The SNPs of delirium and inflammatory factors were harmonized with the MR analysis.

### 2.5. Statistical analysis

The Wald ratio estimator or inverse-variance weighted (IVW) method was used as the primary MR analysis after harmonizing the effect alleles of SNPs associated with inflammatory factors and delirium. If the SNP number was ≥3, the MR-Egger regression and weighted median method were used to test the robustness of the results. The MR-Egger regression and MR-PRESSO method were used to evaluate the possibility of horizontal pleiotropy. In addition to removing the genetic variants of biological evidence of pleiotropy, we also selected genetic variants with a genome-wide significant level (*P* < 5e-6) to include a greater number of SNPs and to improve the statistical power of the analysis.

All analyses in our study were performed using the TwoSampleMR and MR-PRESSO packages in RStudio (2022.12.0+353). This study used a Bonferroni-corrected threshold of a *P*-value of < 0.0006 (≈0.05/9 exposures) as a statistically significant MR result.

## 3. Results

### 3.1. Causal effects between inflammatory factors and delirium

This study evaluated the effect of nine genetically predicted inflammatory factors (TNF-α, CPR, IL-1α, IL-1β, IL-2, IL-6, sIL-6Rα, Soluble gp130, and IL-8) on delirium. The details of the selected instruments for MR analysis are shown in Table 2. The number of SNPs finally varied from 2 to 19 at a liberal significance threshold (*P* < 5e-6). The range of F-statistic was from 20.82 to 4,806.24, indicating that all instruments had a strong potential to predict exposure and can be used for the MR analysis (Burgess and Thompson, 2015).

**Table 2:** Mendelian randomization analysis of biomarkers and delirium.

**Phenotype**	**GWAS data source**	***P***<**5e-8, clumping at** ***r***^**2**^ = **0.001**				***P***<**5e-6, clumping at** ***r***^**2**^ = **0.001**			
		***N***	**Range of** ***F*****-statistic**^#^	**OR (95% CI)**	***P***	***N***	**Range of** ***F*****-statistic**^#^	**OR (95% CI)**	***P***
TNF-α	Suhre et al. (2017)	0	—	—	—	4	21.26–14.93	1.01 (0.83, 1.23)	0.90
CRP	Suhre et al. (2017)	0	—	—	—	2	22.25; 24.11	0.88 (0.68, 1.16)	0.37
	Sun et al. (2018)	4	39.38–52.26	0.86 (0.66, 1.12)	0.26	17	20.92–52.26	1.02 (0.86, 1.21)	0.80
IL-1α	Suhre et al. (2017)	1	31.36	0.85 (0.60, 1.22)	0.38	3	23.23–31.36	0.94 (0.76, 1.16)	0.55
	Sun et al. (2018)	0	—	—	—	19	20.86–26.15	1.02 (0.85, 1.21)	0.83
IL-1β	Suhre et al. (2017)	0	—	—	—	2	21.34; 22.13	0.90 (0.54, 1.51)	0.69
	Sun et al. (2018)	2	34.20; 99.20	1.09 (0.86, 1.37)	0.48	13	21.08–99.20	1.01 (0.86, 1.19)	0.86
IL-2	Suhre et al. (2017)	0	—	—	—	4	21.43–26.60	0.91(0.76, 1.01)	0.36
	Sun et al. (2018)	2	32.88; 60.36	0.99 (0.71, 1.39)	0.95	19	21.03–60.36	1.02 (0.89, 1.18)	0.57
IL-6	Sun et al. (2018)	0	—	—	—	15	20.82–27.20	0.39 (0.27, 0.56)	0.34
	Gilly et al. (2020)	0	—	—	—	14	21.40–27.72	1.03 (0.98, 1.09)	0.23
sIL-6Rα	Suhre et al. (2017)	1	2,377.54	0.89 (0.83, 0.96)	0.0016	3	21.09–2,377.54	0.91 (0.82, 1.01)	0.06
	Sun et al. (2018)	2	65.70; 1,588.92	0.89 (0.79, 0.96)	0.0045	15	20.85–1,588.92	0.94 (0.87, 1.02)	0.13
	Gilly et al. (2020)	2	60.19; 1,106.23	0.88 (0.80, 0.96)	0.0041	16	21.51–1,106.23	0.95 (0.88, 1.03)	0.26
Soluble gp130	Suhre et al. (2017)	1	64.63	1.19 (0.96, 1.47)	0.12	5	34.04–96.37	1.10 (0.95, 1.26)	0.20
	Sun et al. (2018)	4	34.04–96.37	0.98 (0.78, 1.22)	0.83	15	20.84–96.37	0.98 (0.84,1 .14)	0.78
IL-8	Suhre et al. (2017)	0	—	—	—	3	21.14–21.39	1.07 (0.89, 1.28)	0.49
	Sun et al. (2018)	0	—	—	—	17	21.06–28.80	1.06 (0.90, 1.24)	0.50

^#^When less than 3 SNPs were available, all F-statistic values were shown; otherwise, the range of F-statistic range was reported.

Four SNPs were found to be significantly and independently associated with TNF-α at a *P*-value of < 5e-6. No evidence of an association with the risk of delirium was observed in the IVW method (OR = 1.01, 95% CI: 0.83–1.23, *P* = 0.90). Similar results were found using the MR-Egger and weighted median methods (Supplementary Table S1). No pleiotropic effect was detected via the MR-Egger intercept and MR-PRESSO (all *P* > 0.05). The leave-one-out analysis showed that none of the single SNPs substantially affected the overall risk estimation (Supplementary Figure S1).

For CRP, only two SNPs were selected from the Suhre et al. dataset at a *P*-value of < 5e-6 level, and no association with the risk of delirium was observed (OR = 0.88, 95% CI: 0.68–1.16, *P* = 0.37). Given two *P*-value thresholds (5e-8 and 5e-6), we did not observe any strong statistical evidence of an effect on delirium in the Sun et al. dataset. The results of MR-Egger intercept and MR-PRESSO indicated that no SNP has possible pleiotropy (all *P* > 0.05, Supplementary Table S2). The leave-one-out plot showed that the overall estimated effect was not driven by any individual SNP (Supplementary Figure S2).

Regarding IL-1α, only one SNP was extracted from the Suhre et al. dataset, and no SNP was found in the Sun et al. dataset at a *P*-value of < 5e-8. The Wald ratio showed no causal relationship between IL-1α and delirium (OR = 0.85, 95% CI: 0.60–1.22, *P* = 0.38) in Suhre et al. When we set the instrument *P-*value threshold to 5e-6, the results of the MR analysis indicated that there was no causal relationship between IL-1α and the risk of delirium in either the Suhre et al. dataset (OR_ivw_ = 0.94, 95% CI: 0.76–1.16, *P*_ivw_ = 0.55) or the Sun et al. dataset (OR_ivw_ = 1.02, 95% CI: 0.85–1.21, *P*_ivw_ = 0.85). No directional pleiotropy was observed in the MR-Egger intercept or MR-PRESSO (all *P* > 0.05, Supplementary Table S3). The leave-one-out plot showed that the overall estimated effect was not driven by any individual SNP (Supplementary Figure S3).

For IL-1β, no statistical evidence for an association with delirium risk was observed in either the Suhre et al. dataset (OR: 0.94, 95% CI: 0.76–1.16, *P* = 0.55) or the Sun et al. (OR = 1.02, 95% CI: 0.85–1.21, *P* = 0.83) dataset in IVW regression (Supplementary Table S4). The leave-one-out plot showed that the overall estimated effect was not driven by any individual SNP (Supplementary Figure S4).

There was no statistical evidence that higher concentration levels of IL-2 affected the risk of delirium in either the Suhre et al. dataset (OR = 0.91, 95% CI: 0.76–1.11, *P* = 0.41) or the Sun et al. dataset (OR = 1.02, 95% CI: 0.89–1.18, *P* = 0.75) using a significance threshold of a *P*-value of < 5e-6 (Supplementary Table S5). No SNPs were removed due to pleiotropy (Supplementary Figure S5).

For IL-6, the number of SNPs selected from the Suhre et al. dataset at a *P*-value of < 5e-8 and a *P*-value of < 5e-6 was zero. Therefore, we finally used instrumental variants from the Sun et al. and Gilly et al. datasets. The results of the IVW method showed that no effect was observed on the risk of delirium in either Sun et al. dataset (OR = 0.39, 95% CI: 0.27–0.56, *P* = 0.34) or the Gilly et al. dataset (OR = 1.03, 95% CI: 0.98–1.09, *P* = 0.23). Similar results were obtained from the MR-Egger and weighted median methods (both *P* > 0.05, Supplementary Table S6). The results of the MR-PRESSO Global test and MR-egger intercept indicated that no pleiotropy was detected (both *P* > 0.05). The eave-one-out plot showed that the overall estimated effect was not driven by any individual SNP (Supplementary Figure S6).

Regarding sIL-6Rα, when using the Suhre et al. dataset in the primary analysis, only one SNP (*rs*4129267) was significantly and independently associated with sIL-6Rα at a *P*-value of < 5e-8, clumping at *r*^2^ = 0.001. The result of the Wald ratio showed that elevated sIL-6Rα decreased the risk of delirium (OR_waldratio_ = 0.89, 95% CI: 0.79–0.96, *P*_waldratio_ = 0.0016). When using a liberal *P*-value threshold of 5e-6, the results of MR analysis indicated a lack of statistical significance in the relationship between a genetically predicted sIL-6Rα and delirium risk (*P*_IVW_ = 0.06). Similar results were shown in the GWAS data of Sun et al. and Gilly et al. After relaxing the critical value of *P* to 5e-6, we found no strong evidence for sIL-6Rα associated with delirium (Supplementary Table S7). The leave-one-out analysis showed that none of the single SNP substantially affect the overall risk estimation (Supplementary Figure S7).

There was no statistical evidence that higher or lower concentration levels of soluble gp130 increased the risk of delirium in either the Suhre et al. dataset (OR_IVW_ = 1.19, 95% CI: 0.96–1.47, *P*_IVW_ = 0.12) or the Sun et al. dataset (OR_IVW_ = 0.98, 95% CI: 0.78–1.22, *P*_IVW_ = 0.83) at the critical value of *P* of < 5e-8. Findings obtained using a more liberal threshold (*P* < 5e-6) agreed with the previous results. Consistent with IVW, no evidence of the effects of soluble gp130 was obtained in the MR-Egger regression and weighted median methods (Supplementary Table S8). The leave-one-out plot showed that the overall estimated effect was not driven by any individual SNP (Supplementary Figure S8).

Regarding IL-8, no SNPs were extracted at the genome-wide significance level (*P* < 5e-8) in any dataset. When a relaxed significant threshold (*P* ≤ 5e-6) was used, no association with the risk of delirium was observed in either Suhre et al. dataset (OR_IVW_ = 1.07, 95% CI: 0.89–1.28, *P*_IVW_ = 0.49) or in Sun et al. dataset (OR_IVW_ = 1.06, 95% CI: 0.90–1.24, *P*_IVW_ = 0.50). Similar results were also found in the MR-Egger and weighted median methods (Supplementary Table S9). No SNPs were removed due to pleiotropy based on statistical criteria (Supplementary Figure S9).

### 3.2. Reverse analysis

In total, nine SNPs for delirium were identified at the genome-wide significant level (*P* < 5e-6) and LD based on *r*^2^ < 0.001. No significant reverse causal association of delirium on inflammatory factors was detected by IVW or Wald ratio (*P* > 0.05). Detailed information on the reverse analysis is shown in Table 3.

**Table 3:** Reverse analysis of the effects of delirium on the level of inflammatory factors.

**Phenotype**	**GWAS data source**	**Estimate (95% CI)**	***P* for association**	***P* for heterogeneity test**	***P* for MR-Egger intercept**	***P* for MR PRESSO results from the Global Test**
TNF-α	Suhre et al. (2017)^*^	0.35 (−0.09, 0.79)	0.12	—	—	—
CRP	Suhre et al. (2017)^*^	1.40 (0.94, 2.08)	0.10	—	—	—
	Sun et al. (2018)	0.01 (−0.08, 0.10)	0.83	1.54E-07	0.52	0.004
CRP (two outliers-corrected)	Sun et al. (2018)	0.07 (−0.01, 0.15)	0.11	0.26	0.61	0.28
IL-1α	Suhre et al. (2017)^*^	−0.08 (−0.52, 0.36)	0.71	—	—	—
	Sun et al. (2018)	0.98 (0.93, 1.03)	0.38	0.14	0.72	0.30
IL-1β	Sun et al. (2018)	−0.01 (−0.06, 0.04)	0.64	0.13	0.52	0.29
	Suhre et al. (2017)^*^	0.70 (0.45, 1.08)	0.11	—	—	—
IL-2	Sun et al. (2018)	0.007 (−0.03, 0.05)	0.74	0.38	0.54	0.42
	Suhre et al. (2017)	0.82 (0.53, 1.27)	0.37	—	—	—
IL-6	Sun et al. (2018)	1.03 (0.98,1.07)	0.27	0.16	0.45	0.34
	Gilly et al. (2020)	0.97 (0.91, 1.03)	0.40	0.70	0.96	0.71
sIL-6Rα	Suhre et al. (2017)^*^	0.05 (−0.39, 0.49)	0.83	—	—	—
	Sun et al. (2018)	0.03 (−0.02, 0.07)	0.27	0.16	0.45	0.36
	Gilly et al. (2020)	0.01 (−0.05, 0.08)	0.71	0.48	0.87	0.61
Soluble gp130	Suhre et al. (2017)^*^	0.24 (−0.19, 0.68)	0.28	—	—	—
	Sun et al. (2018)	0.03 (−0.02, 0.08)	0.23	0.08	0.76	0.23
IL-8	Suhre et al. (2017)^*^	0.95 (0.62, 1.46)	0.81	—	—	—
	Sun et al. (2018)	1.02 (0.99, 1.06)	0.20	0.79	0.59	0.89

^*^Only one SNP was extracted from the corresponding GWAS dataset, and Wald ratio was used to detect the association. The effect of delirium on other inflammatory factors was identified by inverse-variance weighted.

## 4. Discussion

Delirium is commonly observed among older individuals during their hospitalization and is recognized as a potential risk factor for the subsequent onset of dementia (Goldberg et al., 2020). In this study, we performed bidirectional MR analyses in three population-based datasets to investigate the potential association between peripheral inflammation factors and delirium. No evidence was discovered to support a causal relationship between TNF-α, CRP, IL-1α, IL-1β, IL-2, IL-6, soluble gp130, and IL-8 in the occurrence of delirium, as estimated through various MR analysis methods. There was some evidence that genetically predicted sIL-6Rα was significantly associated with a decreased risk of delirium across all three GWAS data sources at the genome-wide significant level (*P* < 5e-8, *r*^2^ < 0.001). However, the causal relationship became non-significant when a liberal significance threshold (*P* < 5e-6) was employed. Furthermore, there was no evidence to suggest that delirium caused the elevation of these inflammatory biomarkers through genetic variations.

The IL-6 signaling pathway has been extensively investigated in the pathogenesis and progression of delirium. However, the results of these previous studies remain controversial. The study conducted by Noah et al. (2021) revealed no detectable association between IL-6 and delirium in both cardiac and elective non-cardiac surgical procedures, which was consistent with the observations in our study. Conversely, Roh et al. (2022) conducted a meta-analysis that involved 1,107 participants from seven cohort studies and found that patients with POD had significantly higher serum IL-6 levels compared to non-POD patients in the early stages after surgery. Similar results were also observed in Liu et al's. (2018) study, indicating a correlation between the pre-surgery plasma levels of IL-6 and the risk of POD. In addition, previous studies reported that IL-6 exhibited neuroprotective effects when the IL-6/sIL-6Rα trans-signaling pathway was activated (Biber et al., 2008; Ma et al., 2015). The effect may be attributed to the recycling of circulating IL-6 that binds to either the membrane-bound IL-6 receptor or soluble gp130 (Ferreira et al., 2013; Garbers et al., 2014). Westhoff et al. (2013) found that a higher level of IL-6 in the cerebrospinal fluid prior to surgical repair for acute hip fractures was related to a decreased risk of experiencing POD. These findings indicated that the association between IL-6 levels and delirium may be heterogeneous in samples obtained at different time points and locations.

Moreover, findings from our primary analysis at the genome-wide significance level (*P* < 5e-8) indicated that higher sIL-6Rα levels might lower the risk of delirium. However, considering that the limited number of SNPs (less than three) would reduce the statistical power, we set the instrument *P-*value threshold to 5e-6. The causal relationship of sIL-6Rα on delirium became non-significant in all three GWAS data sources. A lack of significant findings in a liberal analysis provides more convincing evidence of the true null of a relationship. This suggests that there is limited evidence supporting a causal connection, even when considering potentially pleiotropic genetic variants in the analysis (Burgess and Thompson, 2015). Consistent with our results, Neerland et al. (2016) reported that the serum levels of CRP, IL-6, and sIL-6R were not significantly associated with POD in patients with or without cognitive impairment before experiencing an acute hip fracture. The role of IL-6/sIL-6Rα in the pathogenesis of delirium is not yet fully understood, and additional research is recommended.

CRP is a pro-inflammatory factor related to increased blood–brain barrier permeability (Hsuchou et al., 2012). A meta-analysis of six studies revealed a correlation between CRP and delirium, but the results presented were highly heterogeneous (*I*^2^ = 72.92%). Another meta-analysis comprising six cohort or case–control studies on CRP found similar results among individuals undergoing cardiac surgery, with no detectable association in either hip fracture or non-cardiac surgery (Noah et al., 2021). With the aid of large-scale GWAS datasets, we did not observe any evidence that genetically elevated CRP was significantly associated with delirium. The lack of association of CRP with delirium in our MR analysis suggests that previous findings from observational studies might be affected by some degree of bias, such as surgical type.

TNF-α, a common pro-inflammatory biomarker, has been considered to be associated with a cognitive decline in patients with Alzheimer's disease (Wang et al., 2023). However, the current investigation yielded no evidence of an effect of TNF-α on the risk of delirium, which was consistent with the results reported by Su et al. (2023). The researchers observed that the concentration of TNF-α in the POD group was elevated on the day following admission to the intensive care unit. However, this statistical significance was lost after accounting for potential confounding covariates, such as demographic variables and intraoperative events. The findings suggested that TNF-α might not function as an initiating factor but rather as a response to the inflammatory cascade during delirium.

Furthermore, several studies have examined the potential role of IL-1α, IL-1β, IL-2, and IL-8 in the development of delirium (Capri et al., 2014; Brattinga et al., 2022; Payne et al., 2023). No consistent conclusion has been drawn. The findings of our study indicated that these biomarkers may not play a central role associated with delirium.

The major strength of our study was employing a two-sample MR design to minimize the risk of reverse causation and residual confounding. Although previous meta-analysis and cohort studies have investigated the associations between inflammatory factors and delirium, the pooling of results from observational studies may be vulnerable to confounding variables that were not originally measured (Noah et al., 2021), such as frailty (Bellelli et al., 2019), health status, use of sedatives (Turan et al., 2020; Mart et al., 2021), analgesic drugs, and degree of anesthesia (Avidan et al., 2017; Fanelli et al., 2022). Therefore, it is not reasonable to establish causal associations. Furthermore, we used sensitivity analyses to reduce pleiotropic concerns and evaluated the causal effects using data from several large-scale GWAS to increase the reliability and consistency of the results.

However, our study had several drawbacks. First, we chose study data in which biomarker concentrations were only evaluated in peripheries rather than cerebrospinal fluid samples. Analyzing inflammatory components in the cerebrospinal fluid would shed more light on the neuroinflammatory process behind delirium. Second, because distinct cytokines can interact in different ways, a series of complementary or opposing interactions can functionally alter these effects on delirium. Third, while all of the genetic instruments utilized in our investigation were effective for MR analysis (*F*-statistics >10), a limited number of genetic variations were used in the majority of cases, which may have reduced statistical power.

## 5. Conclusion

In summary, the MR analysis indicated that there may not be a causal association between delirium and the following factors: TNF-α, CRP, IL-1α, IL-1β, IL-2, IL-6, sIL-6Rα, soluble gp130, and IL-8. Further investigation is required to explore the involvement of inflammatory factors in the pathogenesis of delirium.

## Data availability statement

The original contributions presented in the study are included in the article/Supplementary material, further inquiries can be directed to the corresponding author.

## Author contributions

MY: conceptualization, methodology, data curation, formal analysis, and writing—original draft preparation. YL: data curation, interpretation, and writing—original draft preparation. BL: conceptualization, data curation, supervision, and writing—review and editing. QG: project administration, funding acquisition, and writing—review and editing. All authors read and approved the final manuscript.
