# Supplementary material for: Inflammatory biomarkers and delirium: a Mendelian randomization study
Source: Front Aging Neurosci. 2023 Aug 15;15:1221272. doi: 10.3389/fnagi.2023.1221272 (PMC10464607; doi:10.3389/fnagi.2023.1221272)
Supplement: Supplementary file 1 [file Data_Sheet_1.docx]

Supplementary Material

Article Title

Miao Yu^1†^; Yuxuan Li^2†^; Baohua Li^1*^, Qinggang Ge^2*^

*** Correspondence:**

Qinggang Ge, M.D., qingganggelin@126.com

Baohua Li, M.M., lianglbh@126.com

# Supplementary Methods

*GWAS data for inflammatory factors*

We investigated eight exposures that have been reported to be associated with delirium in previous observational studies (CPR, IL-1α, IL-1β, IL-2, IL-6, sIL-6 Rα, soluble gp130, and IL-8). All studies were selected because the samples restricted to European descent.

TNF-α, CPR, IL-1α, IL-1β, IL-2, sIL-6 Rα, soluble gp130, and IL-8 in plasma associated data were retrieved from the KORA F4 study(Suhre et al., 2017). In this study, Suhre et al. (2017) identified 539 proteins in 3,080 subjects living in Germany. KORA F4 study recruited participants between 2006 and 2008 comprising individuals who, at that time, were aged 32–81 years. A linear model was used to adjust for age, gender and body mass index on inverse normal transformed probe levels.

To further investigate the robustness of the MR results, we also extracted the genetic instruments associated with CPR, IL-1α, IL-1β, IL-2, IL-6, sIL-6 Rα, soluble gp130, and IL-8 from the INTERVAL study(Sun et al., 2018). The INTERVAL study recruited adults with aged 18 years and older from 25 centers of England’s National Health Service Blood and Transplant (NHSBT). Participants were generally in good health and those with a history of myocardial infarction, stroke, cancer, HIV, hepatitis B or C and recent illness or infection were excluded. After genetic quality control, Sun et al. identified 1,487 plasma proteins in 3,301 participants. SOMAscan assay was used to measure the relative concentration of plasma proteins of protein complexes. Protein concentrations are quantified as relative fluorescent units. A linear model on rank-based inverse normal transformed inflammatory factors levels was used to adjust for age, gender, and body mass index.

Regarding IL-6 and sIL-6Rα, in addition to the KORA F4 study and the INTERVAL study, we also obtained the significant and independent genetic variants from Gilly A et al(Gilly et al., 2020). They identified 257 cardiometabolic disease-related serum protein in 1,328 participants from a Greek cohort (MANOLIS). A Linear regression on inverse-normal transformation adjusting for age, age squared, gender, plate number, mean Normalised Protein eXpression level across all proteins, per sample and season.

# Supplementary Figures and Tables

Table S1 Results from TNF-α Mendelian Randomization analyses.

| GWAS source | Methods | OR (95% CI) | *P* for association | *P* for heterogeneity test | *P* for MR-Egger intercept | *P* for MR-PRESSO Global test |
| --- | --- | --- | --- | --- | --- | --- |
| Suhre et al. | *P* < 5e-6, clumping at *r*^2^ = 0.001 (*n* of SNPs= 4) | | | | | |
|  | Inverse variance weighted | 1.01 (0.83, 1.23) | 0.90 | 0.94 |  |  |
|  | MR Egger | 0.76 (0.21, 2.74) | 0.72 | 0.90 | 0.70 |  |
|  | Weighted median | 1.03 (0.81, 1.30) | 0.80 |  |  |  |
|  | MR-PRESSO (raw, 0 outliers) |  |  |  |  | 0.94 |

* The number of SNPs was 0 at the *P* < 5e-8 level.

Table S2 Results from CRP Mendelian Randomization analyses.

| GWAS source | Methods | | OR (95% CI) | *P* for association | *P* for heterogeneity test | *P* for MR-Egger intercept | | | | *P* for MR-PRESSO Global test |
| --- | --- | --- | --- | --- | --- | --- | --- | --- | --- | --- |
| Suhre K et al. | *P* < 5e-6, clumping at *r*^2^ = 0.001 (*n* of SNPs= 2) | | | | | | | | | |
|  | Inverse variance weighted | | 0.88 (0.68, 1.16) | 0.37 | 0.92 | | N/A | | N/A | |
| Sun et al. | *P* < 5e-8, clumping at *r*^2^ = 0.001 (*n* of SNPs= 4) | | | | | | | | | |
|  | Inverse variance weighted | | 0.86 (0.66, 1.12) | 0.26 | 0.60 |  | | | |  |
|  | MR Egger | | 9.09 (0.07, 1161.5) | 0.54 | 0.75 | 0.70 | | | |  |
|  | Weighted median | | 0.89 (0.65, 1.22) | 0.47 |  |  | | | |  |
|  | MR-PRESSO (raw, 0 outliers) | |  |  |  |  | | | | 0.51 |
|  | *P* < 5e-6, clumping at *r*^2^ = 0.001 (*n* of SNPs= 17) | | | | | | | | | |
|  | Inverse variance weighted | 1.02 (0.86, 1.21) | | 0.80 | 0.27 | | |  |  | |
|  | MR Egger | 1.28 (0.74, 2.24) | | 0.39 | 0.26 | | | 0.40 |  | |
|  | Weighted median | 0.98 (0.79, 1.22) | | 0.84 |  | | |  |  | |
|  | MR-PRESSO (raw, 0 outliers) |  | |  |  | | |  | 0.81 | |

* The number of SNPs selected from Suhre K et al was 0 at the *P* < 5e-8 level. N/A, not enough instrumental variables.

Table S3 Results from IL-1α Mendelian Randomization analyses.

| GWAS source | Methods | OR (95% CI) | *P* for association | *P* for heterogeneity test | *P* for MR-Egger intercept | *P* for MR-PRESSO Global test |
| --- | --- | --- | --- | --- | --- | --- |
| Suhre K et al. | *P* < 5e-8, clumping at *r*^2^ = 0.001 (*n* of SNPs= 1) | | | | | |
|  | Wald ratio | 0.85 (0.60, 1.22) | 0.38 | N/A | N/A | N/A |
|  | *P* < 5e-6, clumping at *r*^2^ = 0.001 (*n* of SNPs= 3) | | | | | |
|  | Inverse variance weighted | 0.94 (0.76, 1.16) | 0.55 | 0.70 |  |  |
|  | MR Egger | 0.32 (0.02, 5.18) | 0.57 | 0.71 | 0.59 |  |
|  | Weighted median | 0.91 (0.69, 1.20) | 0.51 |  |  |  |
|  | MR-PRESSO (raw, 0 outliers) |  |  |  |  | N/A |
| Sun et al. | *P* < 5e-6, clumping at *r*^2^ = 0.001 (*n* of SNPs= 19) | | | | | |
|  | Inverse variance weighted | 1.02 (0.85,1.21) | 0.83 | 0.85 |  |  |
|  | MR Egger | 1.23 (0.82, 1.85) | 0.34 | 0.86 | 0.33 |  |
|  | Weighted median | 0.99 (0.77, 1.28) | 0.95 |  |  |  |
|  | MR-PRESSO (raw, 0 outliers) |  |  |  |  | 0.854 |

* The number of SNPs selected from Sun et al. was 0 at the *P* < 5e-8 level. N/A, not enough instrumental variables.

Table S4 Results from IL-1β Mendelian Randomization analyses.

| GWAS source | Methods | OR (95% CI) | *P* for association | *P* for heterogeneity test | *P* for MR-Egger intercept | *P* for MR-PRESSO Global test |
| --- | --- | --- | --- | --- | --- | --- |
| Suhre K et al. | *P* < 5e-6, clumping at *r*^2^ = 0.001 (*n* of SNPs= 2) | | | | | |
|  | Inverse variance weighted | 0.90(0.54,1.51) | 0.69 | 0.13 | N/A | N/A |
| Sun et al. | *P* < 5e-8, clumping at *r*^2^ = 0.001 (*n* of SNPs= 2) | | | | | |
|  | Inverse variance weighted | 1.09 (0.86, 1.37) | 0.48 | N/A | N/A | N/A |
|  | *P* < 5e-6, clumping at *r*^2^ = 0.001 (*n* of SNPs= 13) | | | | | |
|  | Inverse variance weighted | 1.01 (0.86,1.19) | 0.86 | 0.95 |  |  |
|  | MR Egger | 0.99 (0.66, 1.48) | 0.97 | 0.92 | 0.90 |  |
|  | Weighted median | 1.05 (0.85, 1.29) | 0.67 |  |  |  |
|  | MR-PRESSO (raw, 0 outliers) |  |  |  |  | 0.96 |

* The number of SNPs selected from Suhre K et al was 0 at the *P* < 5e-8 level. N/A, not enough instrumental variables.

Table S5 Results from IL-2 Mendelian Randomization analyses.

| GWAS source | Methods | OR (95% CI) | *P* for association | *P* for heterogeneity test | *P* for MR-Egger intercept | *P* for MR-PRESSO Global test |
| --- | --- | --- | --- | --- | --- | --- |
| Suhre K et al. | *P* < 5e-6, clumping at *r*^2^ = 0.001 (*n* of SNPs= 4) | | | | | |
|  | Inverse variance weighted | 0.91 (0.76,1.11) | 0.41 | 0.64 |  |  |
|  | MR Egger | 0.99 (0.46, 2.12) | 0.99 | 0.44 | 0.85 |  |
|  | Weighted median | 0.91 (0.72, 1.14) | 0.36 |  |  |  |
|  | MR-PRESSO (raw, 0 outliers) |  |  |  |  | 0.68 |
| Sun et al. | *P* < 5e-8, clumping at *r*^2^ = 0.001 (*n* of SNPs= 2) | | | | | |
|  | Inverse variance weighted | 0.99 (0.71, 1.39) | 0.95 | 0.42 | N/A | N/A |
|  | *P* < 5e-6, clumping at *r*^2^ = 0.001 (*n* of SNPs= 19) | | | | | |
|  | Inverse variance weighted | 1.02 (0.89,1.18) | 0.57 | 0.59 |  |  |
|  | MR Egger | 0.96 (0.66,1.41) | 0.85 | 0.53 | 0.74 |  |
|  | Weighted median | 1.06 (0.87, 1.29) | 0.74 |  |  |  |
|  | MR-PRESSO (raw, 0 outliers) |  |  |  |  | 0.61 |

* The number of SNPs selected from Suhre K et al was 0 at the *P* < 5e-8 level. N/A, not enough instrumental variables.

Table S6 Results from IL-6 Mendelian Randomization analyses.

| GWAS source | Methods | | OR (95% CI) | *P* for association | *P* for heterogeneity  test | *P* for MR-Egger intercept | | | *P* for MR-PRESSO Global test |
| --- | --- | --- | --- | --- | --- | --- | --- | --- | --- |
| Sun et al. | *P* < 5e-6, clumping at *r*^2^ = 0.001 (*n* of SNPs= 15) | | | | | | | | |
|  | Inverse variance weighted | 0.39 (0.27, 0.56) | | 0.34 | 0.72 | |  |  | |
|  | MR Egger | 0.43 (0.11, 1.65) | | 0.06 | 0.94 | | 0.07 |  | |
|  | Weighted median | 0.45 (0.28, 0.72) | | 0.35 |  | |  |  | |
|  | MR-PRESSO (raw, 0 outliers) |  | |  |  | |  | 0.71 | |
| Gilly et al. | *P* < 5e-6, clumping at *r*^2^ = 0.001 (*n* of SNPs= 14) | | | | | | | | |
|  | Inverse variance weighted | 1.03（0.98，1.09） | | 0.23 | 0.51 | |  |  | |
|  | MR Egger | 1.04 (0.98, 1.01) | | 0.25 | 0.44 | | 0.71 |  | |
|  | Weighted median | 1.02 (0.06, 1.08) | | 0.54 |  | |  |  | |
|  | MR-PRESSO (raw, 0 outliers) |  | |  |  | |  | 0.59 | |

* The number of SNPs selected from Suhre K et al. and Gilly et al. was 0 at the *P* < 5e-8 level.

Table S7 Results from sIL-6Rα Mendelian Randomization analyses.

| GWAS source | Methods | OR (95% CI) | | *P* for association | *P* for heterogeneity  test | *P* for MR-Egger intercept | | *P* for MR-PRESSO Global test |
| --- | --- | --- | --- | --- | --- | --- | --- | --- |
| Suhre K et al. | *P* < 5e-8, clumping at *r*^2^ = 0.001 (*n* of SNPs= 1) | | | | | | | |
|  | Wald ratio | 0.89 (0.83, 0.96) | | 0.0016 |  |  | |  |
|  | *P* < 5e-6, clumping at *r*^2^ = 0.001 (*n* of SNPs= 3) | | | | | | | |
|  | Inverse variance weighted | | 0.91(0.82, 1.01) | 0.06 | 0.12 |  |  | |
|  | MR Egger | | 0.83(0.74,0.94) | 0.20 | 0.32 | 0.32 |  | |
|  | Weighted median | | 0.91(0.85,0.98) | 0.006 |  |  |  | |
|  | MR-PRESSO | |  |  |  |  | N/A | |
| Sun et al. | *P* < 5e-8, clumping at *r*^2^ = 0.001 (*n* of SNPs= 2) | | | | | | | |
|  | Inverse variance weighted | 0.88 (0.79, 0.97) | | 0.008 |  |  | |  |
|  | *P* < 5e-6, clumping at *r*^2^ = 0.001 (*n* of SNPs= 15) | | | | | | | |
|  | Inverse variance weighted | | 0.94 (0.87, 1.02) | 0.13 | 0.94 |  |  | |
|  | MR Egger | | 0.95(0.84, 1.07) | 0.41 | 0.92 | 0.83 |  | |
|  | Weighted median | | 0.95 (0.87, 1.03) | 0.25 |  |  |  | |
|  | MR-PRESSO (raw, 0 outliers) | |  |  |  |  | 0.92 | |
| Gilly et al. | *P* < 5e-8, clumping at *r*^2^ = 0.001 (*n* of SNPs= 2) | | | | | | | |
|  | Inverse variance weighted | 0.88 (0.80,0.96) | | 0.004 | N/A | N/A | | N/A |
|  | *P* < 5e-6, clumping at *r*^2^ = 0.001 (*n* of SNPs= 16) | | | | | | | |
|  | Inverse variance weighted | | 0.95 (0.88, 1.03) | 0.26 | 0.07 |  |  | |
|  | MR Egger | | 0.92 (0.88, 1.03) | 0.21 | 0.07 | 0.44 |  | |
|  | Weighted median | | 0.89 (0.82, 0.98) | 0.01 |  |  |  | |
|  | MR-PRESSO (raw, 0 outliers) | |  |  |  |  | 0.12 | |

* N/A, not enough instrumental variables.

Table S8 Results from soluble gp130 Mendelian Randomization analyses.

| GWAS source | Methods | OR (95% CI) | *P* for association | *P* for heterogeneity test | *P* for MR-Egger intercept | | *P* for MR-PRESSO Global test |
| --- | --- | --- | --- | --- | --- | --- | --- |
| Suhre K et al. | *P* < 5e-8, clumping at *r*^2^ = 0.001 (*n* of SNPs= 1) | | | | | | |
|  | Wald ratio | 1.19 (0.96, 1.47) | 0.12 | N/A | | N/A | N/A |
|  | *P* < 5e-6, clumping at *r*^2^ = 0.001 (*n* of SNPs= 5) | | | | | | |
|  | Inverse variance weighted | 1.10 (0.95, 1.26) | 0.20 | 0.41 | |  |  |
|  | MR Egger | 1.12 (0.73, 1.71) | 0.63 | 0.27 | | 0.92 |  |
|  | Weighted median | 1.12 (0.98, 1.41) | 0.08 |  | |  |  |
|  | MR-PRESSO (raw, 0 outliers) |  |  |  | |  | 0.44 |
| Sun et al. | *P* < 5e-8, clumping at *r*^2^ = 0.001 (*n* of SNPs= 4) | | | | | | |
|  | Inverse variance weighted | 0.98 (0.78, 1.22） | 0.83 | 0.32 |  | |  |
|  | MR Egger | 1.38 (0.66,2.89) | 0.48 | 0.31 | 0.43 | |  |
|  | Weighted median | 0.96 (0.74,1.24) | 0.73 |  |  | |  |
|  | MR-PRESSO (raw, 0 outliers) |  |  |  |  | | 0.38 |
|  | *P* < 5e-6, clumping at *r*^2^ = 0.001 (*n* of SNPs=15) | | | | | | |
|  | Inverse variance weighted | 0.98 (0.84, 1.14) | 0.78 | 0.69 |  | |  |
|  | MR Egger | 1.32 (0.86, 2.04) | 0.23 | 0.79 | 0.17 | |  |
|  | Weighted median | 0.95 (0.76,1.19) | 0.67 |  |  | |  |
|  | MR-PRESSO (raw, 0 outliers) |  |  |  |  | | 0.68 |

Table S9 Results from IL-8 Mendelian Randomization analyses.

| GWAS source | Methods | OR (95% CI) | *P* for association | *P* for heterogeneity test | *P* for MR-Egger intercept | *P* for MR-PRESSO Global test |
| --- | --- | --- | --- | --- | --- | --- |
| Suhre K et al. | *P* < 5e-6, clumping at *r*^2^ = 0.001 (*n* of SNPs= 3) | | | | | |
|  | Inverse variance weighted | 1.07 (0.89, 1.28) | 0.49 | 0.73 |  |  |
|  | MR Egger | 1.03 (0.74, 1.43) | 0.88 | 0.44 | 0.85 |  |
|  | Weighted median | 1.05 (0.84, 1.31) | 0.69 |  |  |  |
|  | MR-PRESSO |  |  |  |  | N/A |
| Sun et al. | *P* < 5e-6, clumping at *r*^2^ = 0.001 (*n* of SNPs=17) | | | | | |
|  | Inverse variance weighted | 1.06 (0.90, 1.24) | 0.50 | 0.42 |  |  |
|  | MR Egger | 1.09 (0.72, 1.64) | 0.69 | 0.35 | 0.87 |  |
|  | Weighted median | 1.09 (0.87, 1.37) | 0.44 |  |  |  |
|  | MR-PRESSO (raw, 0 outliers) |  |  |  |  | 0.42 |

* The number of SNPs selected from Suhre K et al. and Sun et al. was 0 at the *P* < 5e-8 level. N/A, not enough instrumental variables.


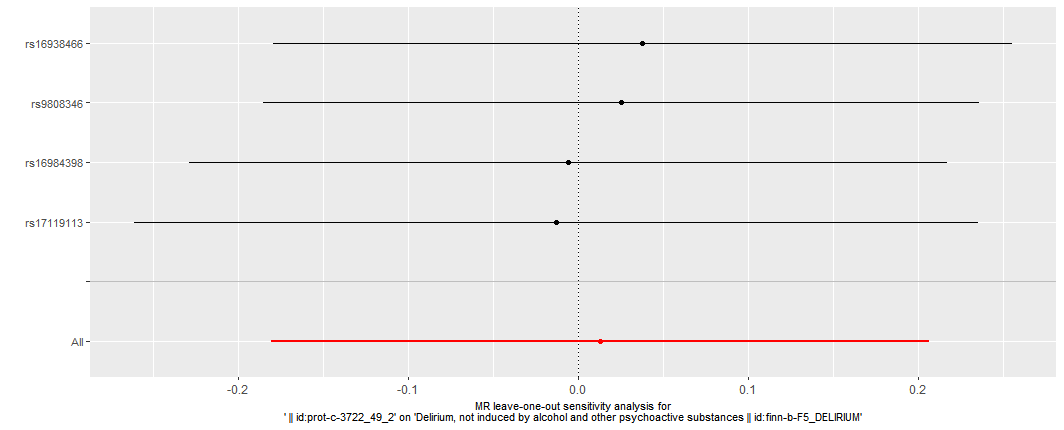


Figure S1. The MR leave-one-out sensitivity analysis for TNF-α on delirium. Each black point represented the inverse variance weighted estimate of TNF-α levels on delirium excluding the individual single nucleotide polymorphism (SNP) on the Y axis. The red point reflected the overall estimate effect including all SNPs. The plot indicated that the overall estimate effect was not driven by any individual SNP. We extracted SNPs associated with TNF-α at the genome-wide significant level (*P* < 5e-6) and linkage disequilibrium clumping approach based on *r*^2^<0.001.


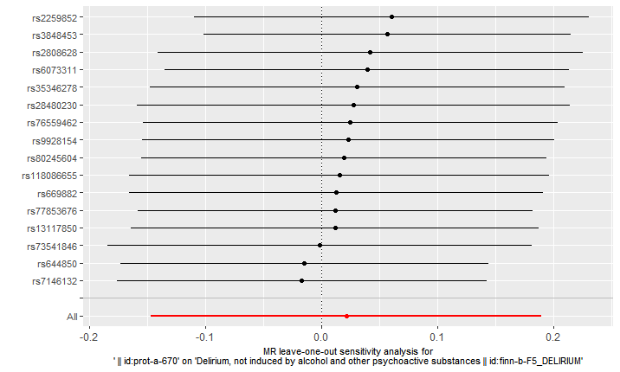


Figure S2. The MR leave-one-out sensitivity analysis for CRP on delirium. Each black point represented the inverse variance weighted estimate of CRP levels on delirium excluding the individual single nucleotide polymorphism (SNP) on the Y axis. The red point reflected the overall estimate effect including all SNPs. The plot indicated that the overall estimate effect was not driven by any individual SNP. The number of SNPs was selected from Sun et al. at a genome-wide significant level (*P* < 5e-6, *r*^2^<0.001). One SNP was removed due to containing missing values by Rstudio, thus only 15 SNPs in the plot. The plot indicated that the overall estimate effect was not driven by any individual SNP.


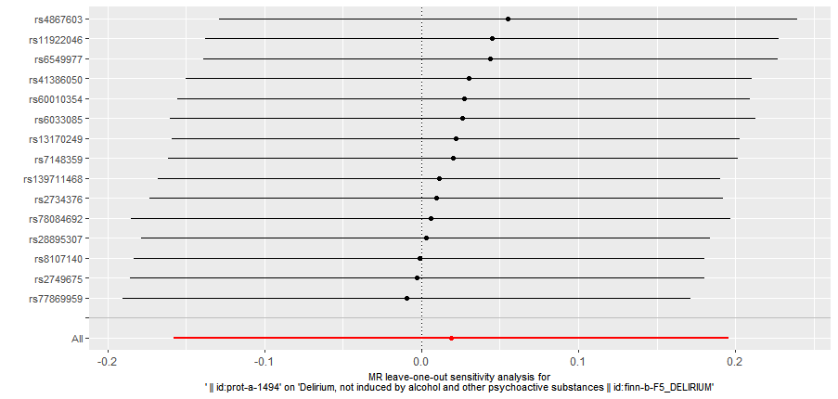

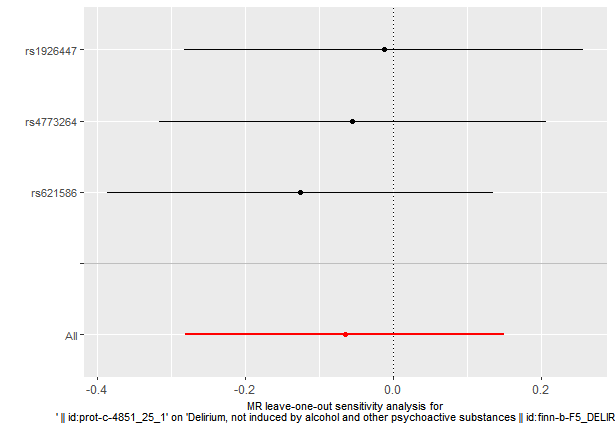
(A) (B)


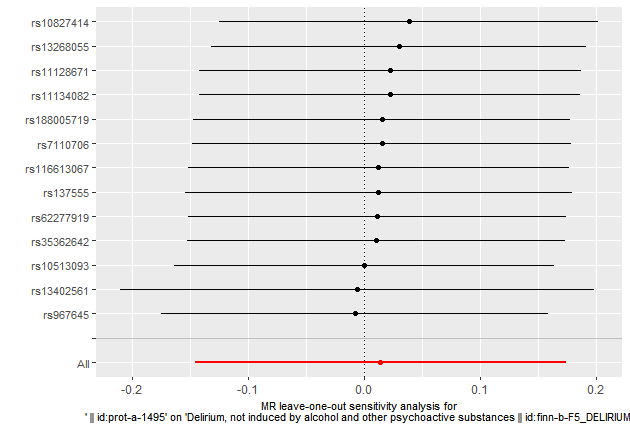
Figure S3. The MR leave-one-out sensitivity analysis for IL-1α on delirium. Each black point represented the inverse variance weighted estimate of IL-1α levels on delirium excluding the individual single nucleotide polymorphism (SNP) on the Y axis. The red point reflected the overall estimate effect including all SNPs. (A) Instrumental variables were selected from Suhre K et al. at a genome-wide significant level (*P* < 5e-6, *r*^2^<0.001). (B) Instrumental variables were selected from Sun et al. at a genome-wide significant level (*P* < 5e-6, *r*^2^<0.001). Four SNPs was removed due to containing missing values by Rstudio, thus only 15 SNPs in the plot. The plot indicated that the overall estimate effect was not driven by any individual SNP.

Figure S4. The MR leave-one-out sensitivity analysis for IL-1β on delirium. Instrumental variables were selected from Sun et al. at a genome-wide significant level (*P* < 5e-6, *r*^2^<0.001). The plot indicated that the overall estimate effect was not driven by any individual SNP.

(A) (B)


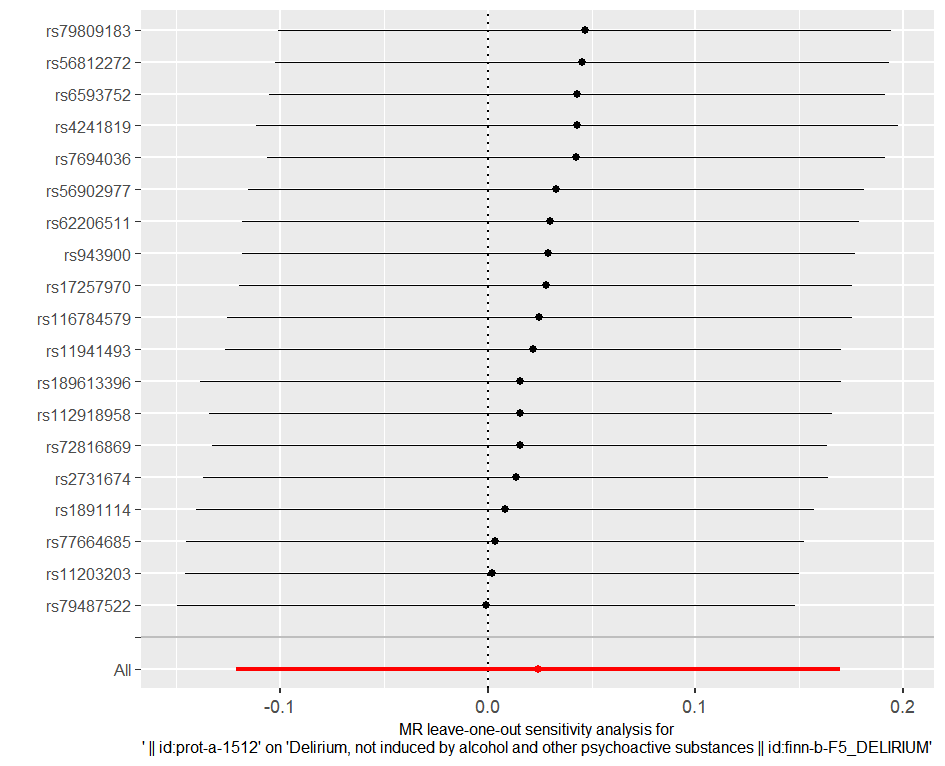

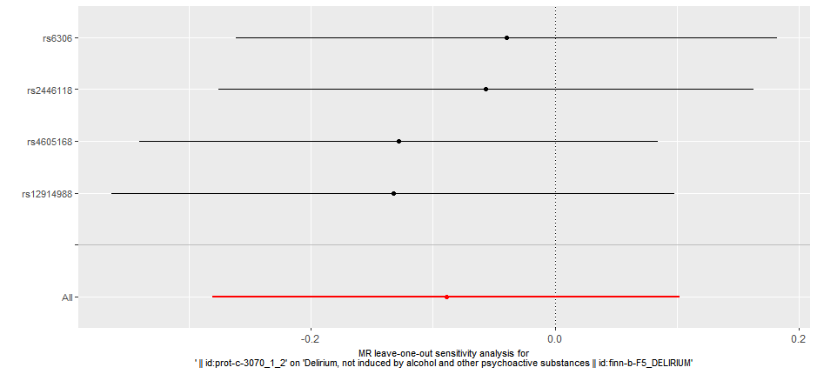
Figure S5. The MR leave-one-out sensitivity analysis for IL-2 on delirium. Each black point represented the inverse variance weighted estimate of IL-2 levels on delirium excluding the individual single nucleotide polymorphism (SNP) on the Y axis. The red point reflected the overall estimate effect including all SNPs. (A) Instrumental variables were selected from Suhre K et al. at a genome-wide significant level (*P* < 5e-6, *r*^2^<0.001). (B) Instrumental variables were selected from Sun et al. at a genome-wide significant level (*P* < 5e-6, *r*^2^<0.001). The plot indicated that the overall estimate effect was not driven by any individual SNP.


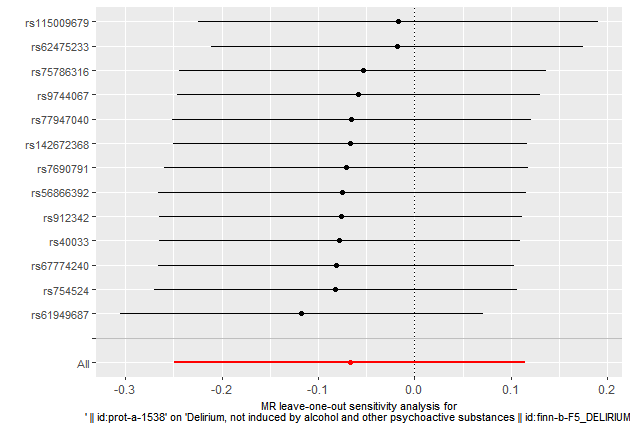

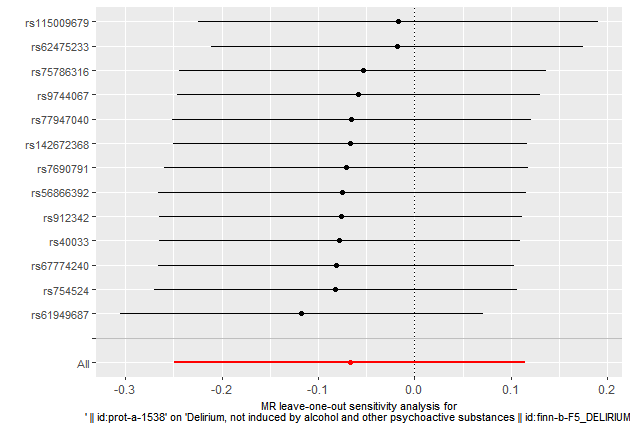
(A) (B)

Figure S6. The MR leave-one-out sensitivity analysis for IL-6 on delirium. Each black point represented the inverse variance weighted estimate of IL-2 levels on delirium excluding the individual single nucleotide polymorphism (SNP) on the Y axis. The red point reflected the overall estimate effect including all SNPs. (A) Instrumental variables were selected from Sun et al. at a genome-wide significant level (*P* < 5e-6, *r*^2^<0.001). Two SNPs was removed due to containing missing values by Rstudio, thus only 13 SNPs in the plot. (B) Instrumental variables were selected from Gilly et al. at a genome-wide significant level (*P* < 5e-6, *r*^2^<0.001). One SNP was removed due to containing missing values by Rstudio, thus only 14 SNPs in the plot. The plot indicated that the overall estimate effect was not driven by any individual SNP.


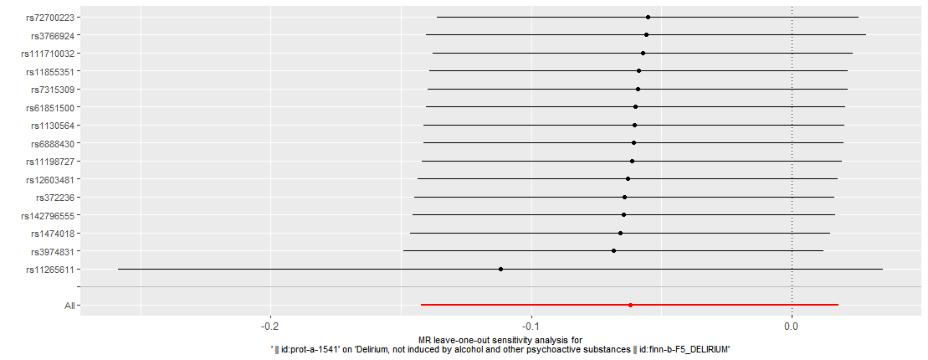

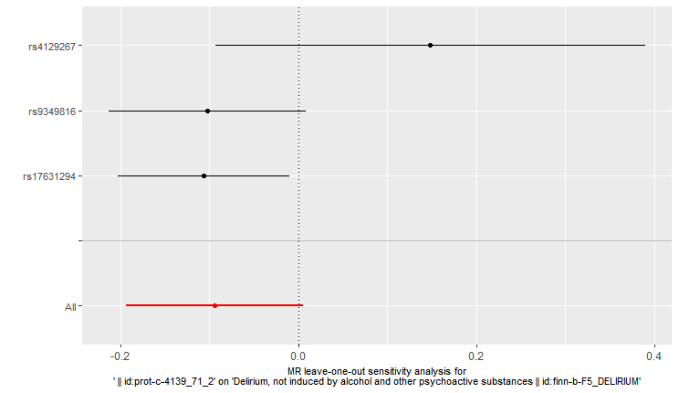
(A) (B)


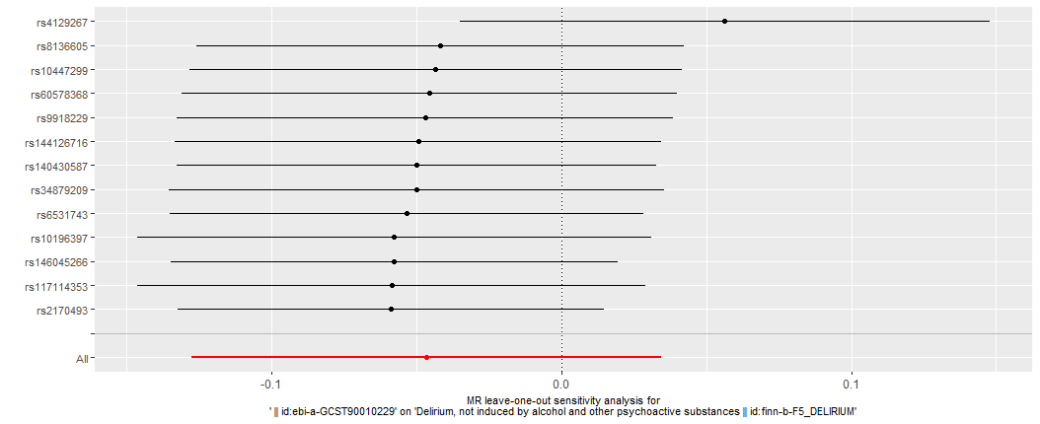
(C)

Figure S7. The MR leave-one-out sensitivity analysis for sIL-6α on delirium. Each black point represented the inverse variance weighted estimate of sIL-6α levels on delirium excluding the individual single nucleotide polymorphism (SNP) on the Y axis. The red point reflected the overall estimate effect including all SNPs. (A) Instrumental variables were selected from Suhre K et al. at a genome-wide significant level (*P* < 5e-6, *r*^2^<0.001). (B) Instrumental variables were selected from Sun et al. at a genome-wide significant level (*P* < 5e-6, *r*^2^<0.001). (C) Instrumental variables were selected from Gilly et al. at a genome-wide significant level (*P* < 5e-6, *r*^2^<0.001). Three SNPs was removed due to containing missing values by Rstudio, thus only 13 SNPs in the plot. The plot indicated that the overall estimate effect was not driven by any individual SNP.


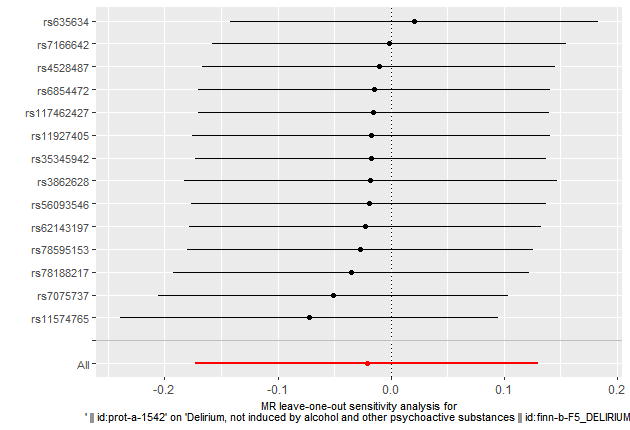

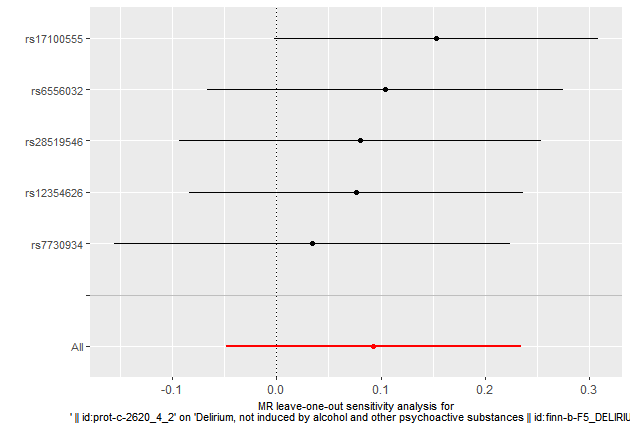
 (A) (B)

Figure S8. The MR leave-one-out sensitivity analysis for gp130 on delirium. Each black point represented the inverse variance weighted estimate of gp130 levels on delirium excluding the individual single nucleotide polymorphism (SNP) on the Y axis. The red point reflected the overall estimate effect including all SNPs. (A) Instrumental variables were selected from Suhre K et al. at a genome-wide significant level (*P* < 5e-6, *r*^2^<0.001). One SNP was removed due to containing missing values by Rstudio, thus only four SNPs in the plot. (B) Instrumental variables were selected from Sun et al. at a genome-wide significant level (*P* < 5e-6, *r*^2^<0.001). One SNP was removed due to containing missing values by Rstudio, thus only 14 SNPs in the plot. The plot indicated that the overall estimate effect was not driven by any individual SNP.


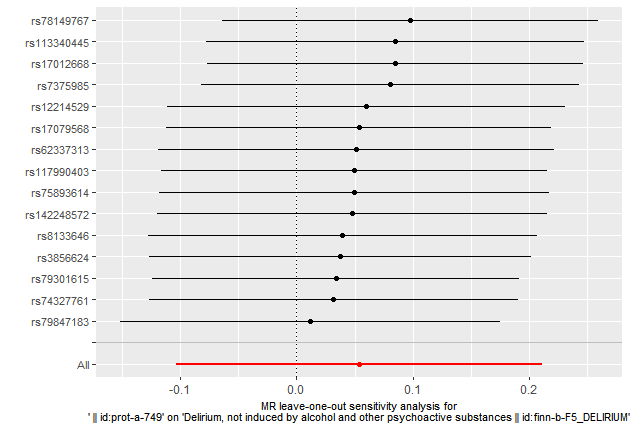

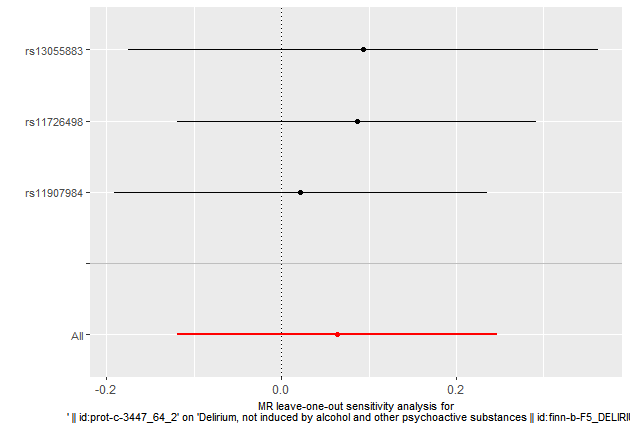
 (A) (B)

Figure S9. The MR leave-one-out sensitivity analysis for IL-8 on delirium. Each black point represented the inverse variance weighted estimate of IL-8 levels on delirium excluding the individual single nucleotide polymorphism (SNP) on the Y axis. The red point reflected the overall estimate effect including all SNPs. (A) Instrumental variables were selected from Suhre K et al. at a genome-wide significant level (*P* < 5e-6, *r*^2^<0.001). (B) Instrumental variables were selected from Sun et al. at a genome-wide significant level (*P* < 5e-6, *r*^2^<0.001). Two SNPs was removed due to containing missing values by Rstudio, thus only 15 SNPs in the plot. The plot indicated that the overall estimate effect was not driven by any individual SNP.

# Reference

Gertow, K., Sennblad, B., Strawbridge, R. J., Ohrvik, J., Zabaneh, D., Shah, S., et al. (2012). Identification of the BCAR1-CFDP1-TMEM170A locus as a determinant of carotid intima-media thickness and coronary artery disease risk. *Circ Cardiovasc Genet, 5*(6), 656-665. doi:10.1161/CIRCGENETICS.112.963660

Gilly, A., Park, Y. C., Png, G., Barysenka, A., Fischer, I., Bjornland, T., et al. (2020). Whole-genome sequencing analysis of the cardiometabolic proteome. *Nat Commun, 11*(1), 6336. doi:10.1038/s41467-020-20079-2

Suhre, K., Arnold, M., Bhagwat, A. M., Cotton, R. J., Engelke, R., Raffler, J., et al. (2017). Connecting genetic risk to disease end points through the human blood plasma proteome. *Nat Commun, 8*, 14357. doi:10.1038/ncomms14357

Sun, B. B., Maranville, J. C., Peters, J. E., Stacey, D., Staley, J. R., Blackshaw, J., et al. (2018). Genomic atlas of the human plasma proteome. *Nature, 558*(7708), 73-79. doi:10.1038/s41586-018-0175-2
